# Faster and targeted raster imaging through non-rectangular scanning

**DOI:** 10.1107/S1600576726008277

**Published:** 2026-09-15

**Authors:** Frederik H. Gjørup, Thorbjørn E. K. Christensen, Yimeng Li, Marcelo J. P. Alcocer, Henrik Birkedal, Innokenty Kantor, Mads R. V. Jørgensen

**Affiliations:** a Department of Chemistry and iNANO Aarhus University Langelandsgade Aarhus 8000 Denmark; b MAX IV Laboratory Lund University Fotongatan 2 221 00 Lund Sweden; c Department of Applied Mathematics and Computer Science Technical University of Denmark Richard Petersens Plads, Build. 324 Kongens Lyngby 2800 Denmark; d Department of Physics Technical University of Denmark Fysikvej, Build. 310 Kongens Lyngby 2800 Denmark; Montanuniversität Leoben, Austria

**Keywords:** scanning diffraction, microbeam X-ray diffraction, XRD imaging, synchrotron radiation

## Abstract

Many materials are spatially heterogenous and it is important to characterize this heterogeneity, which can be done by scanning microbeam X-ray diffraction (µXRD) or X-ray fluorescence (µXRF) microscopy. A modified approach to raster scanning is presented, where each line in a raster scan is of varying length, allowing for efficient scanning of non-rectangular areas. This shortens the measurement time significantly and similarly reduces the large amount of data generated from the area outside the sample that is normally produced for non-rectangular samples. The scanning procedure has been implemented for µ-X-ray diffraction and fluorescence mapping at the DanMAX diffraction beamline at MAX IV, Sweden. It was found that the reduction in required measurement time was nearly proportional to the reduction in scanned area, a significant reduction for non-rectangular samples. As an example, a section of the dactyl club of a stomatopod specimen (*Odontodactylus scyllarus*) was scanned.

## Abstract 2

A highly efficient algorithm for raster scanning an area of non-rectangular shapes is presented. This method can significantly reduce the required scanning time for *e.g.* microbeam X-ray diffraction and X-ray fluorescence scanning microscopy.

## 1. Introduction

Raster scanning, the generation of images through point-wise acquisition of data over a larger grid, is a widespread technique finding uses in many fields of science as a means to produce spatially resolved images. Common examples in materials science characterization include scanning probe techniques, such as atomic force microscopy, magnetic force microscopy and scanning tunnelling microscopy, as well as scanning electron microscopy techniques, scanning spectroscopy and scanning X-ray diffraction microscopy.

There are many examples of raster imaging techniques utilizing synchrotron radiation, such as nano-X-ray-fluorescence mapping (Falcones *et al.*, 2025 ▸; Vajda *et al.*, 2025 ▸), scanning transmission X-ray microscopy (Feggeler *et al.*, 2023 ▸; Butcher *et al.*, 2025 ▸; Plouviez, Guieysse, Buwalda *et al.*, 2024 ▸; Plouviez, Guieysse, Wolmarans *et al.*, 2024 ▸), combined X-ray fluorescence and diffraction mapping with texture analysis (Rodriguez-Palomo *et al.*, 2026 ▸; Christensen *et al.*, 2025 ▸), small-angle X-ray microscopy (SAXS) (Leu *et al.*, 2016 ▸; Rinnerthaler *et al.*, 1999 ▸), and 3D techniques such as X-ray diffraction computed tomography (XRD-CT) and tensor tomography, to name a few (Grünewald *et al.*, 2024 ▸; Rodriguez-Palomo *et al.*, 2024 ▸; Beale *et al.*, 2014 ▸; Liebi *et al.*, 2015 ▸; Bleuet *et al.*, 2008 ▸; Stock *et al.*, 2008 ▸; Meirer & Weckhuysen, 2018 ▸).

Conventionally, raster scan imaging is performed on a rectangular grid, with each ‘pixel’ containing a full dataset for the respective technique, whether it be a single value per pixel (height, absorption, force *etc*.) or multiple values (spectrum, interference pattern, diffraction pattern *etc*.). While a regular rectangular grid is often convenient from a practical perspective, it can waste precious time and resources if a significant part of the measured grid is empty or not of interest due to a non-regularly shaped sample or region of interest. Here, a varying line alternative to rectangular raster scanning is introduced, where the start and end point of each line in the raster grid is specified, allowing significant reduction in measurement time for non-rectangular samples. The approach has been developed and implemented for scanning microbeam X-ray diffraction (µXRD) and fluorescence (µXRF) microscopy at the DanMAX beamline at the MAX IV synchrotron, Sweden, but could easily be applied to a broad range of raster scanning methods. To the best of our knowledge, descriptions of such non-rectangular raster scanning techniques have not been published.

The approach relies on an initial conventional rectangular raster scanning to perform a coarse, *i.e.* low-spatial-resolution, overview map. Such coarse scans can be quite fast; for instance, a 10 by 10 mm area scanned with 200 by 200 µm steps at 50 Hz might take about 4 min. These data are used to select an irregularly shaped region of interest (ROI) for a high-resolution line-wise raster scan. The overview scan allows quick and intuitive selection of the ROI based on the signal of the probing technique in question (powder diffraction in the present case), thus not relying on borders that are visible to the human eye. This leads to a reduction in measurement time that is nearly proportional to the ratio of the rectangle area and the inscribed ROI area (excluding the time required for the overview scan and selection of the ROI). As an example, the measurement time for a perfect circle could be reduced by nearly 21% and that of a triangle by nearly 50%; see examples in the supporting information Fig. S1. For more examples of scan durations, see Table S1; samples with various shapes were scanned, showing an average time reduction of ∼45% across all scans, including the time for the overview scan.

While the measurement and segmentation of the coarse map add to the total spent time, the tasks can be somewhat parallelized by performing the segmentation step of one sample while the measurement of the coarse map of the next sample is ongoing, significantly reducing the added overhead when several samples are available side by side.

**Figure 1:**
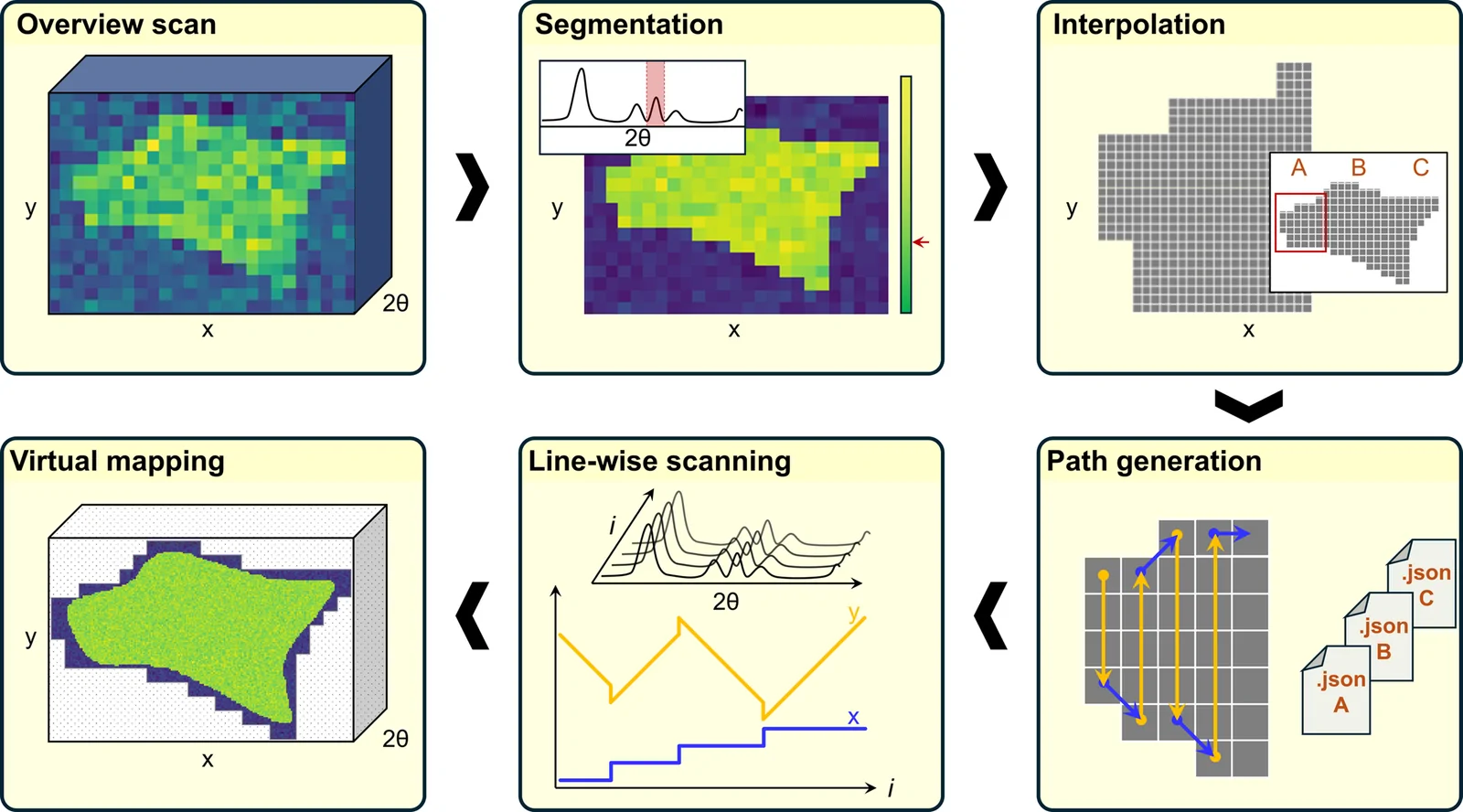
Flowchart of the line-wise scanning procedure for a scanning powder diffraction microscopy image. The following steps are involved: A low-spatial-resolution overview raster scan is measured. Data are reduced to a 2D image which is then subjected to threshold segmentation. This is followed by high-spatial-resolution interpolation and determination of start and end points for each line, and optional splitting into smaller subregions. Scan parameters for each subregion are saved to .json files. High-resolution measurements are conducted as a continuous-motion line-wise scan with synchronized detector and encoder readouts for each point. Finally, data are remapped to virtual HDF5 datasets for visualization.

A simplified flowchart of the line-wise scanning procedure for a scanning µXRD microscopy image is outlined in Fig. 1 ▸. First, a quick, low-spatial-resolution overview raster scan is measured (typically taking a few minutes). The diffraction data of each pixel are reduced to a 2D image according to the signal/peak of interest. The ROI is selected from the 2D image using *e.g.* threshold segmentation and/or manual drawing. The ROI is then interpolated to the desired resolution, before the start and end points for each individual line are found. The scan parameters for each line (start point, end point, number of points and acquisition time) are saved to a .json file, which serves as instructions for the measurement macro. Each line is scanned in a continuous motion (‘flyscan’) with synchronized detector and encoder readouts for each point along the line. The lines are scanned in alternating directions, such that the end point of the previous line is close to the start point of the next line. The measured data are initially saved as ‘flat’ arrays in chronological order, thus excluding any points outside the ROI, but can be saved as virtual HDF5 datasets such that the spatial information is preserved for visualization. In practice at the DanMAX beamline, the segmentation, interpolation and .json file writing are handled in an in-house-developed graphical user interface (GUI), an example of which is available in the supporting information Fig. S2, along with an example of a .json file in Fig. S3. The source code for the GUI is open and available from GitHub (Gjørup, 2026 ▸); though developed for the DanMAX file formats and structure, minor modifications would allow general use at other facilities. The current implementation expects each line to be continuous from start point to end point, and any ‘holes’ in the ROI are filled out by the GUI. For disconnected regions within the same ROI, separate command .json files are made for each continuous region.

Reducing the scan to only include the area of interest leads to a reduction in measurement time, which in turn allows more samples to be measured within the allocated time and affords a much more efficient use of beam time. It also reduces the data quantities, requiring less data storage, and makes subsequent data analysis easier and more efficient. The reduction in measured data can be particularly valuable for high-resolution data of multi-dimensional techniques, such as X-ray powder diffraction with large area detectors, where each pixel comprises a several megabyte raw image. Cropping the data collection to exclude areas otherwise masked during subsequent data processing can speed up the analysis and make more advanced analysis techniques more viable, such as peak fitting, Rietveld refinement, and texture and strain analyses, just to name a few relevant to powder diffraction.

The primary reduction in scan duration going from a rectangular to a non-rectangular map comes from the reduction in the number of points along each line. The longer the lines (in number of points), the larger the relative gain. The overhead associated with changing line (acceleration/deceleration and repositioning) is similar to that of rectangular scans but can vary slightly from line to line, due to the variation in distance between the end point of one line and the start point of the next line. For fast scan rates and/or short line lengths, the line change overhead starts to dominate and the relative reduction in scan duration diminishes. An analytical estimate of the efficiency is presented in the supporting information S1.

As a demonstration of this approach, we here present µXRD and µXRF microscopy imaging of a section of the dactyl club of a *Odontodactylus scyllarus* stomatopod specimen, a species of mantis shrimp known for its powerful club-like appendage capable of punching with an acceleration comparable to that of a bullet (Patek *et al.*, 2004 ▸; Patek & Caldwell, 2005 ▸). The toughness of the dactyl club has been linked to the hierarchical macro-, micro- and nanostructure, combining biominerals and polymers in several distinct regions of the club (Weaver *et al.*, 2012 ▸; Chua *et al.*, 2021 ▸). The club was first scanned with µXRD without continuous motion (Weaver *et al.*, 2012 ▸), and later with higher resolution through flyscanning (Christensen *et al.*, 2023 ▸; Chua *et al.*, 2021 ▸). Here we continue this development, with optimized raster scanning. µXRD microscopy mapping provides clear contrast between the crystalline phases present in the different regions of the club, while µXRF microscopy mapping provides elemental contrast for any heavier elements (heavier than Ar in the present implementation). Furthermore, texture information (crystallite orientation) in the scanning plane can be obtained pixel-wise from the azimuthal intensity variation of the diffraction rings. The dactyl club data presented here are meant as an example of how simple data analysis and even model-free approaches can yield insights. The example is not intended to draw new conclusions regarding the biology and structure of the dactyl club; instead, we refer to existing literature.

## 2. Methods

### 2.1. **µ**XRD and **µ**XRF microscopy

µXRD and µXRF microscopy imaging were performed at the DanMAX beamline, MAX IV, Sweden. A monochromatic beam of 17 keV focused with compound refractive lenses to an elliptical beam shape of approximately 36 µm horizontal by 22 µm vertical (FWHM) was used. Diffraction data were recorded with a DECTRIS PILATUS3 X 2M CdTe area detector at a sample-to-detector distance of 212 mm. The diffraction images were azimuthally integrated to 1D and 2D (180 bins) diffraction patterns using the *MatFRAIA* (Jensen *et al.*, 2022 ▸) algorithm implemented in the beamline live integration pipeline. Fluorescence spectra were measured with a single-element RaySpec SiriusSD silicon drift detector and a Quantum Detectors Xspress3 mini pulse processor, placed in a backscattering position in the horizontal plane. The sample was raster scanned on the *x* axis (horizontal, slow) and *y* axis (vertical, fast), perpendicular to the *z* axis (beam direction), using two OWIS LIMES 84N-270 stages with two-phase step motors and linear encoders with 50 nm encoder resolution.

A coarse rectangular overview scan was measured with 200 by 200 µm steps. A 10 by 10 times higher resolution line-wise selected area raster scan was performed with 20 by 20 µm steps at 20 Hz. The measured area corresponded to 64% of a 299 × 283 rectangle (54136 pixels out of 84617).

### 2.2. Software and scanning orchestration

The scans were orchestrated by *Sardana* (Coutinho *et al.*, 2011 ▸). *Sardana* interacts with the *Tango* control layer (Chaize *et al.*, 1999 ▸; Juerges *et al.*, 2023 ▸) used at MAX IV and many other synchrotrons. *Sardana* sets up each scan line in both the IcePAP motor controller (Janvier *et al.*, 2013 ▸) and a PandABox (Zhang *et al.*, 2017 ▸). The IcePAP controller governs the motion of the fast axis in closed-loop mode, ensuring that the stage is moving at a constant speed before reaching the first trigger (encoder) position. The PandABox simultaneously reads the same encoder signal and is used to record encoder position, shutter control and detector triggering. Knowing the experimental shutter’s opening time, we open it as close to the first scan point as possible, ensuring that the shutter is fully open before the first exposure. During the line scan, the detectors are triggered using TTL signals from the PandABox, which also records the encoder positions at the beginning and end of each exposure. In addition, we record the mean encoder position for each exposure, which is used in the data analysis. Deceleration and shift of the line happen after the last scan point in the line has been completed.

### 2.3. Dactyl club sample preparation

A mantis shrimp of the species *Odontodactylus scyllarus* was acquired from an aqua­rist vendor in Stockholm, Sweden, and sacrificed on arrival in Aarhus, Denmark. It was stored in 50% ethanol for one day, followed by 70% ethanol. The right club was subsequently isolated from the ethanol-preserved specimen. See Christensen *et al.* (2023 ▸) for the full sample preparation description.

The dactyl club sample corresponds to ‘club 5’ in the previously published paper by Christensen *et al.* (2023 ▸). The sample was previously exposed to X-rays in the course of the Christensen *et al.* (2023 ▸) study, both at the DanMAX beamline and with a laboratory X-ray tomography instrument.

### 2.4. Analysis of scanning microscopy data

Non-negative matrix factorization (NMF) was used to segment the µXRD microscopy data into four principal components, using the Python-based algorithm available from *scikit-learn* (Pedregosa *et al.*, 2011 ▸). Diffraction data were used in a *Q* range of 0.57–3.73 Å^−1^ for all non-zero pixels in the µXRD microscopy image. The pixel-wise non-zero weights of components 1–3 were [0–1] normalized and combined to an RGB composite. The four components are shown in supporting information Fig. S4.

The XRF energy was calibrated using the elastic peak and the Ca *K* peak present in the sample. The XRF spectra were fitted using *pyMCA* (Solé *et al.*, 2007 ▸) with a custom batch fitter. The major components were Ca, Sr, Br, Zn and Ni. The relative contents of Ca, Sr and Br obtained from the pixel-wise batch fitting were combined to an RGB composite image.

## 3. Results and discussion

From segmentation of the coarse overview scan of the mantis shrimp dactyl club section, the measured area of the line-wise raster scan map was reduced to 64% of a 299 × 283 rectangle of equal resolution (54136 pixels out of 84617). The scan took just over 42 min, whereas the full rectangle would have taken approximately 75 min to measure (bi-directional), corresponding to an approximately 40% reduction in scanning time. Adding 10 min for the overview scan and segmentation gives a net reduction of approximately 30%.

**Figure 2:**
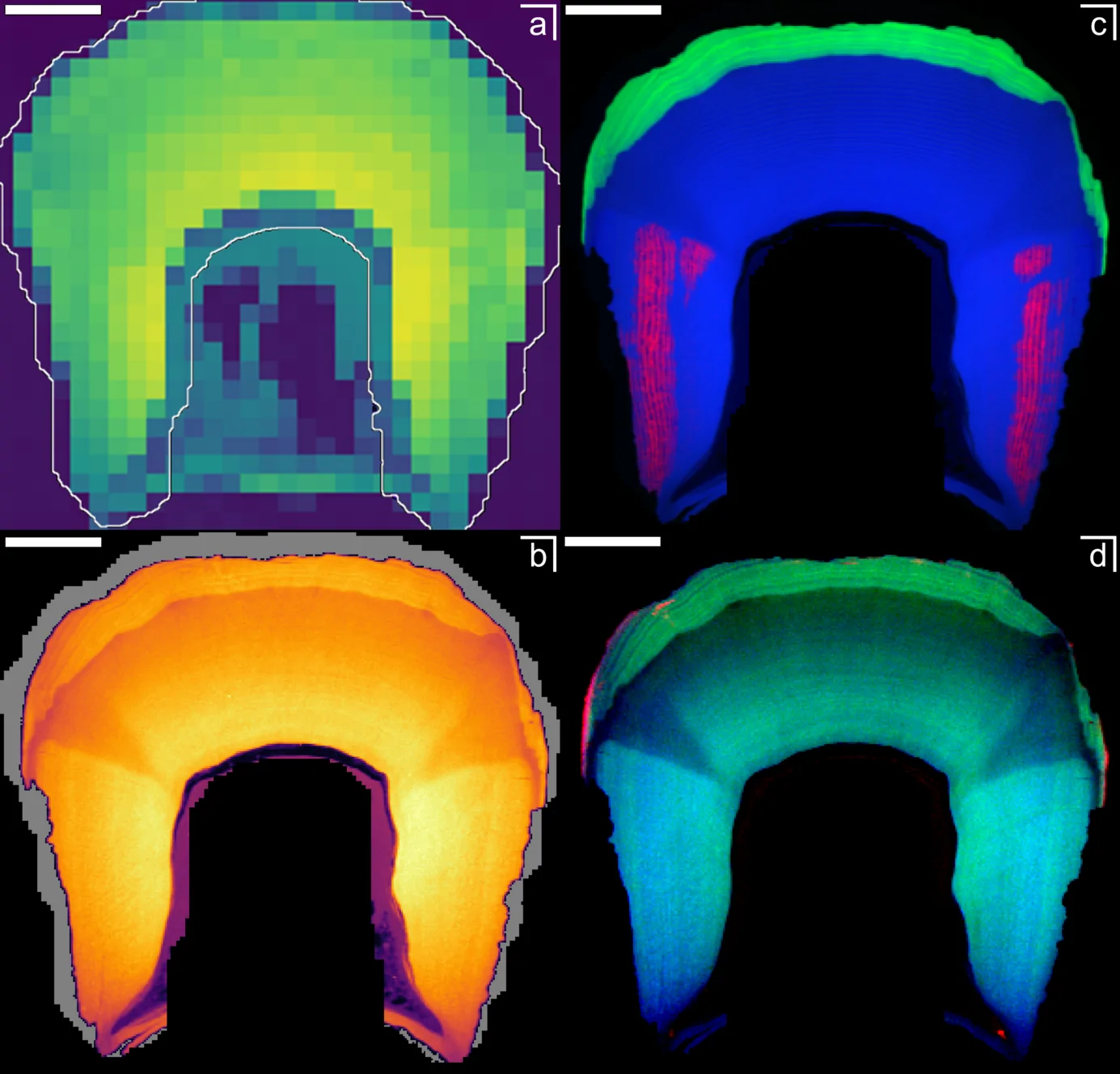
(*a*) Overview scan (average diffraction signal). (*b*) Total fluorescence signal (1–18 keV, extending 1 keV above incident energy to include full elastic peak). (*c*) NMF segmentation (red: calcite rich; green: HAp rich; blue: chitin rich), [0–1] normalized for each RGB channel. (*d*) Relative fluorescence (*K*-edge; red: Br; green: Sr; blue: Ca). All scale bars are 1 mm.

The combination of µXRD and µXRF microscopy imaging provides great insight into the spatial distribution of both crystalline phases and elemental composition of the investigated sample. The information from both techniques can be analysed in many ways, ranging from very simple qualitative approaches to detailed quantitative approaches. Four such examples with increasing complexity are shown for a mapped section of the mantis shrimp dactyl club in Fig. 2 ▸. The image in Fig. 2 ▸(*a*) is an example of the low-resolution overview scan, used for the segmentation to determine the high-resolution ROI. The coarse overview scan is shown here as the average diffraction signal for each pixel, but any arbitrary range of the diffraction signal could be used to discriminate in the segmentation. The superimposed white outline shows the actual ROI used in the high-resolution scan.

Fig. 2 ▸(*b*) shows the total fluorescence signal, summed over the 1–18 keV range (extended 1 keV above the incident X-ray energy to include the full elastic peak). The area in grey shows pixels that were included in the line-wise scan but have a negligible fluorescence signal. As the dactyl club section is <100 µm thick, the total fluorescence signal can be considered a pr­oxy for the X-ray absorption and thus the density of the sample.

Fig. 2 ▸(*c*) shows a segmentation obtained from NMF of the X-ray diffraction data using four components, presented as an RGB correlation map. The four components could be identified as a calcite-rich component (red), a hy­droxy­apatite (HAp)-rich component (green), a chitin-rich component (blue) and a ‘background’ component (omitted), in agreement with previous studies which also found amorphous calcium carbonate and amorphous calcium phosphate to be the main components of the dactyl club (Weaver *et al.*, 2012 ▸; Christensen *et al.*, 2023 ▸). The NMF map is a great example of a model-free segmentation method that requires very little prior knowledge of the system and can be generated immediately after data acquisition, serving as a starting point for continued analysis. NMF can also be used directly on the coarse overview scan to define regions for high-resolution imaging. The HAp-rich region shown in green is the impact region at the front of the dactyl club, while the red region with a high calcite content is at the sides of the club. The chitin-rich blue component can be seen throughout the club, but more so where the two other components are less present. This is in agreement with previous observations on the same sample by Christensen *et al.* (2023 ▸). NMF, like many other multivariate algorithms, *e.g.* singular value decomposition, principal component analysis and multivariate curve resolution–alternating least squares, can be immensely useful on its own or in combination with other analysis strategies, such as Rietveld refinement (Birkbak *et al.*, 2017 ▸).

Fig. 2 ▸(*d*) is a composite RGB correlation map of the relative fluorescence (*K* edge), comparing Br (red), Sr (green) and Ca (blue). Note how µXRF demonstrates that the entire club is Ca rich while µXRD shows that only the upper periodic and parts of the side regions contain crystalline phases (HAp and calcite, respectively), the remaining calcium being present in amorphous phases. This highlights the usefulness of correlative µXRD and µXRF analyses and indeed multimodal techniques in general (Grünewald *et al.*, 2024 ▸).

The usefulness of the presented measurement strategy ultimately relies on the precision and reproducibility of the scanning stages, as well as accurate position encoders to ensure reliable positioning of the sample and mitigate motor backlash. In the present example, the scan resolution is 20 µm, while the linear motor encoders have a significantly smaller sub-micrometre resolution. By controlling the motors in closed-loop mode, issues caused by motor backlash are completely eliminated. In general, when designing a bi-directional scanning assembly, it is necessary to have a linear encoder directly measuring the sample position to avoid motion backlash. The encoder must additionally have an appropriate resolution that is significantly smaller than the intended scan resolution. The resolution and reproducibility of the motor stages and encoders used at the DanMAX beamline have been tested experimentally and are confirmed to be sub-micrometre. We recommend generally confirming the performance of translation hardware, in particular for bi-directional scanning.

## 4. Conclusion

Here, we demonstrate an efficient approach to raster scanning non-rectangular samples, providing a faster and more targeted measuring strategy than conventional rectangular scanning. The method relies on scanning the area line by line while changing the start and end points of each line to follow the shape of the sample closely. This approach significantly saves scanning time for most samples and reduces the amount of data and deposited dose outside the region of interest correspondingly.

We have presented an example of µXRD and µXRF imaging of a biological sample and shown examples of the information that can be extracted from such experiments. The approach has been implemented at the DanMAX beamline at MAX IV but is applicable for all scanning microscopy techniques where non-rectangular samples are imaged. We also believe this could be an interesting approach for other scanning techniques, such as XRD-CT of non-cylindrical objects (Leemreize *et al.*, 2013 ▸; Birkbak *et al.*, 2015 ▸; Wittig *et al.*, 2019 ▸; Grünewald *et al.*, 2024 ▸; Rodriguez-Palomo *et al.*, 2024 ▸; Meirer & Weckhuysen, 2018 ▸; Beale *et al.*, 2014 ▸) and in particular SAXS/WAXS tensor tomography (Grünewald *et al.*, 2020 ▸; Grünewald *et al.*, 2023 ▸; Liebi *et al.*, 2015 ▸), where the gains afforded by scanning only areas of interest will be even greater owing to the higher number of scanning dimensions. Note that for 3D techniques using tomographic reconstruction, full completeness of the angular projections is required, which must be accounted for in the initial overview and segmentation to avoid partial volumes.

## Data Availability

Data are available upon request. The segmentation GUI source code is available on GitHub (Gjørup, 2026 ▸); test data are available upon request. *Sardana* scanning macros are available upon request.
